# The Efferent System or Olivocochlear Function Bundle – Fine Regulator and Protector of Hearing Perception

**Published:** 2010-12

**Authors:** Raphael Richard Ciuman

**Affiliations:** *Department of Otorhinolaryngology, Head and Neck Surgery, Marienhospital Gelsenkirchen, Virchowstr. 122, Gelsenkirchen, Germany*

**Keywords:** olivocochlear bundle, medial efferent system, lateral efferent system, superior olive, tinnitus, neurotransmitter

## Abstract

The efferent system of the ear possesses several distinct functions, in particular noise protection, mediation of selective attention and improvement of signal to noise ratio. It also supports adaptation and frequency selectivity by modification of the micromechanical properties of outer hair cells. There are many differences in anatomy and physiology between the medial and lateral olivocochlear system suggesting that they are functionally separate systems. The efferent system is affected by inner ear stressors, e.g. noise, ototoxic drugs, and might play a key role in tinnitus generation and maintenance. The anatomy, physiology and its realtionships to inner ear pathologies are discussed in this review article.

## INTRODUCTION

In 1946, Grant Rasmussen reported his discovery of the olivocochlear system, and since then auditory scientists have been trying to understand how this system exactly works (1). Commonly accepted are relationships to diseases of the auditory sytem and specific main functions including noise protection on the one hand and mediation of selective attention and improvement of signal to noise ratio on the other hand. The efferent system also supports adaptation and frequency selectivity by modification of the micromechanical properties of outer hair cells. Consequently, the lateral and medial efferent system together form the basis for localization of a sound stimulus and enable to function in a three-dimensional auditory world. Terminology distinguishes between the medial and lateral efferent system and the crossed and uncrossed efferent system, respectively. Various neurotransmitters are involved in the subtle mechanisms of fine regulation of the efferent system ensuring above mentioned functions.

### Anatomical characterization

**Cerebral origins and course:** The lateral efferent system originates from the lateral superior olive (LSO) and the medial efferent system from the periolivary region (medial, ventral and anterior) around the medial superior olivary (MSO) complex and the trapezoid body (2) (Table 1). In human there is no nucleus trapezoid body and the lateral efferent component is relatively small compared with other species. But the lateral system still seems to be the largest portion of the mammalian efferent system, with larger size in high-frequency hearing animals (3–5). In contrast, the medial superior olivary nucleus reflects a steady increase in primates corresponding to the capability of low-frequency hearing (6). The well developed human medial olivary nucleus seems to be the basis for extraction of interaural time and phase differences, whereas the smaller human lateral olivary nucleus probably functions in analysis of interaural differences in frequency and intensity. The lateral and medial nuclei together form the basis for localization of a sound stimulus and enable us to function in a three-dimensional auditory world (7, 8).

**Table 1:** Comparison of the medial and lateral efferent system

Medial efferent system	Lateral efferent system

origin fom periolivary region around the medial superior olive	origin from lateral superior olive
medial efferent collaterals project near the afferent projections of type 2 spiral ganglion cells and the peripheral regions of the VCN, subpeduncular granule cells and nucleus Y	lateral system collaterals overlap extensively with type1 spiral ganglion cell afferent input and central regions of the VCN (ventral cochlear nucleus
innervates the inner ear contralateral and ipsilateral	projects mainly ipsilateral
myelinated in the internal auditory canal until exit through the habenula perforata	unmyelinated in internal auditory canal
fibers continue to run in the tunnel spiral bundle, and to a less extent at the floor of the tunnel of Corti as outer spiral fibers together with the type2 spiral ganglion cell peripheral processes and directly innervate the outer hair cells	correspond to the inner spiral bundle and innervate the dendrites of radial afferent fibers under inner hair cells
neurotransmitter include ACh, GABA, CGRP, ATP, enkephalins and NO	neurotransmitter include ACh, GABA, CGRP, dopamine, serotonin, and opioids like dynorphin or enkephalin
synapses of the medial system are formed earlier in development than these of the lateral system and degenerate more slowly after the axons are cut	
more terminals are localised in the basal or mid cochlea	extent of lateral efferent terminals is uniform ipsilateral and stronger at the apex contralateral
high frequency hearing	low frequency hearing
modification of interaural time and phase differences	modification of interaural frequency and intensity

**Figure 1:**
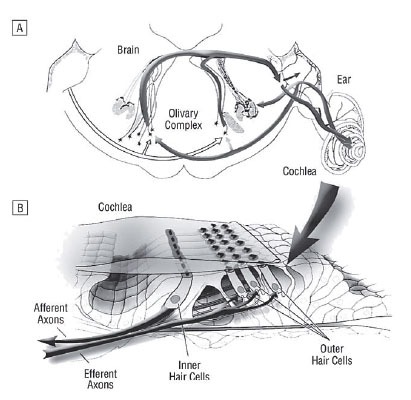
Course of the medial and lateral efferent system. A, The auditory brainstem section. Sound representations from the ear ascend to the olivary complex via the ventral afferent pathway and project back to the ear via dorsal crossed and uncrossed medial and lateral efferent fibers. B Cross-sectional view of the inner ear. The major ascending afferent pathway arises from inner hair cells. Descending olivocochlear projections terminate on inner and outer hair cells. (with permission from Liberman MC. Effects of chronic cochlear de-differentiation on auditory-nerve response. Hear Res 1990; 49: 209–224, © 1990, Elsevier; and May BJ, Budelis J, Niparko JK. Behavioral studies of the olivocohlear efferent system. Arch Otolaryngol Head Neck Surg 2004; 130: 660–664; Copyright © 2004, American Medical Association. All rights reserved).

In the lateral superior olive the descending and the ascending neurons are intermingled (Figure 1). The lateral superior olivary nucleus shows two types of olivocochlear neurons. The small ones (intrinsic neurons) run in the inner spiral bundle and terminate in one or two dense patches with no more than 10–20% over the cochlea length. The large or shell neurons show a more diffuse projection and extend over 50% of the organ of Corti length, and as a group course in the inner spiral bundle at least 80%, but sometimes 95% of the total cochlear length. The large neurons branch and travel 1–2 mm beneath the inner hair cells, forming numerous en passant swellings and a few branches en route shown in various animal experiments (9). Functionally, delay and chopper neurons within the lateral superior olive can be distinguished. Chopper neurons are characterized by a regular repetitive firing pattern with a short and precise latency, what may be attributed to a large extent to their membrane properties (10).

Complex neural processing is found in the spiral ganglion and ventral cochlear nucleus (VCN). Lateral system collaterals overlap extensively with type1 spiral ganglion cell afferent input and central regions of the VCN. Medial efferent collaterals project near the afferent projections of type2 spiral ganglion cells and the peripheral regions of the VCN, subpeduncular granule cells and nucleus Y (11).

The posteroventral cochlear nucleus (PVCN) possesses efferent projections to the medial and lateral olivary structure (12, 13), and the medial and lateral olivocochlear nerves send collaterals to the cochlear nuclei as well (14). A lesion in the PVCN, but not in the anteroventral (AVCN) or dorsal (DCN) subdivisions produces permanent disruption of the medial olivocochlear (MOC) reflex, that affects sound processing and offers protection from acoustic overstimulation. This supports the thesis that some PVCN neurons project to MOC neurons. Here the chopper units rather than the input units represent the MOC reflex interneurons. The most likely pathway of the MOC reflex for sound protection seems to be: hair cells→type1 nerve fibers→PVCN chopper units→MOC neurons→MOC terminates on outer hair cells (15).

**Inner ear course and efferent terminals:** There exists an exchange of nerve fibers between the cochlear nerve and the superior and inferior vestibular nerve within the internal auditory canal (16). The medial and lateral efferent fibers are supposed to run within the inferior vestibular nerve, only joining the cochlear nerve at the anastomosis of Oort, a bundle of 1300 fibers running from the saccular branch of the inferior vestibular nerve to the cochlear nerve (17). The efferent fibers enter the cochlea with the auditory nerve, travel through Rosenthal’s canal, and the medial efferent fibers become unmyelinated as they exit the canal through the habenula perforata. In contrast, the lateral efferent fibers are unmyelinated the whole pathway. The synapses of the medial efferent system are formed earlier in development than these of the lateral system, and degenerate more slowly after the axons are cut.

The medial efferent system innervates the inner ear contralateral and ipsilateral, whereas the lateral efferent system projects mainly ipsilateral (18). The fibers of the lateral efferent system mainly correspond to the inner spiral bundle and innervate the dendrites of radial afferent fibers under inner hair cells, whereas the fibers of the medial efferent system continue to run in the tunnel spiral bundle, and to a less extent at the floor of the tunnel of Corti as outer spiral fibers together with type2 spiral ganglion cell peripheral processes. The medial efferent fibers directly innervate the outer hair cells (19, 20) (Figure 1). To a lesser extent, they also form synapses on afferent and efferent fibers (21).

In the rat, the afferent-efferent fiber-ratio is 7:1 on inner hair cells contrasting with a 1:2 ratio on outer hair cells (22). The efferents on inner hair cells are smaller, more numerous and densely packed than endings on outer hair cells. More medial efferent terminals are localised in the basal or mid cochlea representing a sensitivity correlative, whereas the extent of lateral efferent terminals is uniform ipsilateral and stronger at the apex contralateral (19). A radial gradient exists from the first to the third row of outer hair cells (23). Outer hair cells in the first row receive a disproportionately large number of efferent boutons, relative to other rows, and this effect increases in apical areas. The staining pattern in the cochlear apex starts to decline from and the decreasement is strongest at the outer hair cell rows (24). This contrasts with the increasing size of the afferent neurons and hair cells in the more apical regions. Large efferent fibers are localised to a higher extent at the base and in the first row of outer hair cells and small fibers show the opposite pattern (25). Large efferent fibers beneath outer hair cells, that are rich in neurotubules and other cytoplasmic organelles, decrease from base to apex corresponding with the frequency selectivity at the basilar membrane. In contrast, the small fibers possess maxima at the base and at the apex and the minima corresponds to the frequency range of maximum sensitivity (22).

The intense synaptic activity involving inner hair cells and both afferent and efferent tunnel fibers, at their crossroad, implies functional connections between inner and outer hair cells in the process of hearing (26). There is evidence for efferent synapses onto outer spiral fibers and onto outer hair cell efferents, especially as they cross the tunnel in the tunnel spiral bundle (27, 28).

### Physiological characterization

**Neurotransmitter of the medial and lateral efferent bundle:** Neurotransmission of the efferent system takes place by inhibitory and excitatory transmitters reflecting fine regulation and noise protection (Table 2). The excitatory glutamatergic afferent transmission of the auditory system is under inhibitory control of GABA and dopamine, whereas afferent dendrites can be excited via muscarinic receptors as well (29). The neurotransmitter of the medial olivocochlear fibers include ACh (acetylcholine), GABA (gamma aminobutyric acid), CGRP (calcitonin gene related peptide), ATP (adenosine triphosphate), enkephalins and nitric oxide (NO) (30, 31). The transmitter of the lateral efferent system include ACh, GABA, CGRP, dopamine, serotonin, and opioids like dynorphin or enkephalin. Neurotransmitter can be co-localized, e.g. ACh immunoreactivity can be co-localized with CGRP and opioid-immunoreactivity, and the different types of opioids can be co-localized in the lateral olivocochlear neurons as well (32–34). It was proposed that the neurotransmitters ACh, dynorphine and CGRP selectively lower the cochlear ‘set point’ or resting potential and thereby enhance neural activity. On the other hand, inhibitory neurotransmitters like GABA, dopamine and enkephalin raise the set point of the cochlea, thereby decreasing cochlear activity (35). Consequently, the numerous neurotransmitters provide for the auditory system a wide operating range to enhance or depress environmental stimuli.

**Table 2:** Function and physiological/anatomical correlatives of the medial and lateral efferent system

Functional aspect	Anatomical / Physiological correlative

Noise protection	Activation of nicotinic-like ACh-receptors (nAChRs) induces hyperpolarization of the hair cell membrane and a reduction of afferent firing;
	Activation of acetylcholine alpha 9/alpha 10 receptors (ACh 9/10) at the synapse between efferents and outer hair cells leads to calcium entry into the hair cell, thus inducing a hyperpolarizing Ca^2+^-sensitive K^+^ current, mediated by small conductance channels (Isk), what hyperpolarizes the cell membrane and thus changes the resting potential and the gain of the cochlear amplifier;
	GABAA receptors associated chloride channels in the postsynaptic outer hair cell membrane mediate hyperpolarization and elongation of the cell;
	Hyperpolarizarion causes expansion of prestin molecules, which elongate the outer hair cells;
	Dopamine agonists reduce cochlear damage by noise or ischemia;
	Dopaminergic lateral olivocochlear efferents drive a permanent gain control of the site of auditory action potential initialization; dysfunction represents an early sign of exitoxicity.
Improvement of signal to noise ratio	Improvement in speech in noise intelligibility during contralateral broad band noise application / contralateral acoustic stimuli enhances speech perception, when ipsilateral signal to noise ratio is 10 dB or 15 dB;
	Excitatory neurotransmitters like ACh, dynorphine and CGRP selectively lower the cochlear ‘set point’ and thereby enhancing neural activity; inhibitory neurotransmitters like GABA, dopamine and enkephalin raise the set point of the cochlea, thereby decreasing cochlear activity→the numerous neurotransmitters provide for the auditory system a wide operating range to enhance or depress environmental stimuli;
	Broadband signals, like those present in natural environments, are among the most effective in stimulating the activity of the medial efferent system.
Adaptation to sound	ACh in cochleobasal outer hair cells reduces the stiffness of the lateral wall, but increases the regulatory stiffness response and stretch induced slow cell motility; GABA effects the outer hair cell membrane qualitatively similar cochleoapical;
	Olivocochlear neurons require 50 to 500 ms of stimulation before they respond→efferents bring transient responses to brief speech-like pulses out of the adapting and/or suppressed background noise.
Frequency selectivity regulation	More medial efferent terminals are localised in the basal or mid cochlea/extent of lateral efferent terminals is uniform ipsilateral and stronger at the apex contralateral;
	Dopaminergic olivocochlear neurons are almost exclusively seen in the medial high frequency limb of the lateral superior olive and in the first two turns of the cochlea-selective modulation of high frequency fibers;
	Crossed olivocochlear efferents reduce the receptor potentials on inner hair cells predominantly at the point of highest frequency selectivity;
	Frequencies of 1000-4000 Hz have the highest suppression effect in contralateral acoustic stimulation;
	Section of the efferent bundle decreases frequency selectivity, as an enlargement of the tip segment of the CAP tuning curve can be found and the Q10 dB value decreases by about 30% without any significant threshold change in outer hair cells.
Mediation of selective attention	Selective attention increases the amplitude of EOAEs to the respective ear when attention is directed to that side;
	Patients with an impaired efferent system have a reduced ability to focus attention in the frequency domain and detect signals at unexpected frequencies better than before.
Functionality in a three dimensional auditory world/localization of sound/speech restoration	Noisy, relatively broadmand signals, like those present in natural environments, are among the most effective in stimulating the activity of the medial efferent system;
	The lateral efferent system is supposed to produce a range of set-points, generating a continuum of spontaneous activities and sensitivities, which in turn provides a greater dynamic range for the driven activity of the auditory nerve.
	Medial efferent system is supposed to play a role in intensity discrimination in dichotic noise in humans, as the ILD are reduced, when contralateral noise is added, and what appears to be significantly correlated to the contralateral EOAE amplitude attenuation effect speech restoration of fragmented words or sentences is reliant on olivocochlear bundle function.

The inner spiral bundle shows highest inhibitory GABA-ergic innervation in the basal half in animals (36). In contrast, GABA-ergic innervation and GABAA-receptors on outer hair cells are higher expressed in the apex than at the base of the cochlea (37–39). AChE (acetylcholinesterase) and CGRP are expressed higher basal than apical and stronger in the first outer hair cell row than in the third (40, 41).

ACh in cochleobasal outer hair cells reduces the stiffness of the lateral wall, but increases the regulatory stiffness response and stretch induced slow cell motility. This effect is qualitatively similar to GABA cochleoapical and dependent on extracellular calcium, what could be the base for an influence on adaptation (42). GABA probably by GABAB receptors increases the intracellular calcium and inhibite glutamate response in spiral ganglion neurons (43). The GABAA receptor associated chloride channel in the postsynaptic outer hair cell membrane allows hyperpolarization and elongation of the cells (44).

Nicotinic-like Ach-receptors induce a hyperpolarization of the hair cell membrane leading to a reduction of afferent firing, whereas muscarinic-like receptors induce both a hyper- and depolarization of the plasma membrane (45). The alpha9 subunit of the nicotinic alpha 9/10 ACh-receptor possesses mixed muscarinic and nicotinic properties (46). The expression of the alpha9 subunit is proportional to the efferent system strength. Consequently, the inter-animal variability may be one mechanism contributing to the inter-animal variability in acoustic injury (47). This receptor is stronger expressed in outer than in inner hair cells and only few in the spiral ganglion (48). At the synapse between efferents and outer hair cells the receptor mediates calcium entry into the hair cell, thus inducing a hyperpolarizing Ca^2+^-sensitive K^+^ current, mediated by small conductance channels (Isk), what hyperpolarizes the cell membrane and thus changes the resting potential and the gain of the cochlear amplifier (49–51). In mammalian ear, this leads to a reduction in basilar membrane motion, altering auditory nerve fiber activity and reducing the dynamic range of hearing (52). It was found that ACh also effects the stiffness of the membrane-bound motor protein prestin, that is presumed to be responsible for the electromotile response, and other lateral wall stiffness components (53). The hyperpolarizarion causes expansion of the prestin molecules, which elongates the outer hair cells. Therefore, the medial efferent system may act in “refle” fashion by changing the cochlear amplifier as a consequence of the amount of auditory pathway activity and may also act to provide protection from overstimulation by noise (54). There are additional actions of ACh on the outer hair cell, through other receptor mechanisms, including a “slow effect”, that may use a second messenger system and influence intracellular calcium pools (55–58), and calcium dependent K^+^ channels (59–61). Intracellular pathways involving the GTPases (guanosine triphosphatase) RhoA, Rac1 and Cdc42 may regulate outer hair cell motility (62).

It was shown that the alpha9/alpha10-receptors can be inhibited by the opioids dynorphin (kappa agonist) und endomorphin1(mu agonist), but not enkephalin (delta agonist) (63).

CGRP has a wide expression in the cochlear and vestibular efferent system. The CGRP fibers are stronger expressed on inner than outer hair cells (64) and the staining pattern on outer hair cells mimic with AChE (65). In human, the neurons expressing both ACh and CGRP comprise 35-50% of the total number of efferents (8).

Serotonin is probably expressed in the medial and lateral efferent system as well, and could represent a projection of the reticular formation on the auditory receptor (66). Plasmamembrane serotonin transporters are present in cochlear serotonergic fibers below inner and outer hair cells (67). The highly particularly pattern of serotonin together with the lack of response to sound stimulation suggest that serotonergic fibers constitute cochlear innervation (68).

Dopaminergic olivocochlear neurons were almost exclusively seen in the medial high frequency limb of the lateral superior olive and in the first two turns of the cochlea what may represent a correlative of selective suppression of high frequency fibers (69). The dopaminergic lateral olivocochlear efferents drive a permanent gain control of the site of auditory compound action potential (CAP) initialization. Their dysfunction represents an early sign of exitoxicity (70). It was found that dopamine agonists reduce cochlear damage by noise or ischemia (71–73). It was shown that this transmitter may protect hair cells in inner ear stress, e.g. ischemia (74). Dopamine can depress the activated firing rate by of afferent neurons via dopamine 1 (D1) and dopamine 2 (D2) receptor subtypes, but shows a slight effect on the spontaneous firing rate (75).

NO positive nerve endings were found in the inner spiral bundle and beneath inner and outer hair cells (76, 77).

ACh, Dynorphin and CGRP can lower the resting potential or set-point and potentiating the action potential of glutamate in achieving depolarization and increasing auditory nerve activity. In contrast, GABA, dopamine and enkephalin raise the resting potential and make the peripheral processes they influence less sensitive to glutamate activation by inner hair cells (75, 78–80). The function of the lateral olivocochlear system may therefore be to produce a range of set-points, generating a continuum of spontaneous activities and sensitivities, which in turn provides a greater dynamic range for the driven activity of the auditory nerve (81). An additional lateral efferent loop may allow the dynamic range to be adapted to different levels of activation and besides it might also provide noise protection (71).

**Physiological correlations:** As well as the whole auditory system, the efferent system has a greater right-sided activity in young right-handed adults, but this effect decreases with age and age-related hearing loss (82, 83). Olivocochlear bundle function as the whole auditory system function seems to be susceptible to strenghthening by training as it could be shown that efferent suppression is stronger in musicians (84). Training might be in particular important for patients in hearing loss as efferent function is important for comprehension of acoustically-distorded speech (85). Loss of efferent feedback is expected to degrade perception in noise, as animals with lesioned olivocochlear bundle exhibited significantly elevated thresholds for stimulus location when tested in background noise (86). The auditory efferents are involved in antimasking and complex processing in noisy environments (87). The role of the efferent system in antimasking is supported by the fact of an improvement of speech in noise intelligibility during contralateral broad band noise application (87). It could be shown that contralateral acoustic stimuli enhances speech perception, when ipsilateral signal to noise ratio was 10 dB or 15 dB, and this enhancement had significant positive correlation with contralateral suppression of OAEs (88).

**Otoacoustic emissions (OAEs) measurements:** The olivocohlear bundle plays an inhibitory role on the activity of outer hair cells. Its stimulation reduces auditory nerve response, basilar membrane motility and OAEs amplitude. Due to presence of the crossed olivocochlear bundle, an ipsilateral stimulation of efferent fibers results in both ipsi- and contralateral response. Collet *et al*. observed that otoacoustic emissions in humans can be suppressed by contralateral white noise (89) and OAEs supression after contralateral auditory stimulation seems to be the only objective and none invasive method for evaluation of the functional integrity of the medial efferent system and of the structures lying on its course. Selective attention increases the amplitude of EOAEs to the respective ear when attention is directed to that side (90, 91) and section of the olivocochlear bundle abolishes the inhibitory effect on OAEs in contralateral stimulation (92, 93).

The contralateral suppression of transient evoked otoacoustic emissions (TEOAEs) is present in 88.5% of neonates (94). Preterm neonates show reduced spontaneous otoacoustic emissions (SOAEs) in contrast to full-term neonates (95). The suppression of EOAEs is dependent on stimulus frequency and intensity, is greatest when the suppressor and the studied EOAEs have similar frequencies and can be investigated with broad-band noise, narrow-band noise, pure tones or clicks. White noise and pure tones of 1000 to 2000 Hz have the greatest suppressor effect on TEOAEs (96). For amplitude-modulated tones, it could be shown that for suppression the intensities have to be greater than 40 dB and the greater the modulation depth, the greater the suppression effect - with significant effect for 75-100% modulation depth (97).

Medial olivocochlear (MOC) reflex: The most likely pathway of the MOC reflex for sound protection seems to be: hair cells→type1 nerve fibers→PVCN chopper units→MOC neurons→MOC terminates on outer hair cells. Mammals that lack the medial efferent innervation of outer hair cells demonstrate either extreme specialization for high-frequency (distinct bat species) (98–101) or low-frequency (blind mole rat) (102). The MOC bundle attenuates the response of the cochlea to sound by reducing the gain of the outer hair cell mechanical response to stimulation. The MOC system probably functions in a protective role by acting to reduce receptor damage during intense acoustic exposure. In natural environments the system could function as a mechanism for “unmasking” biologically significant acoustic stimuli by reducing the response of the cochlea to simultaneous low-level noise (103). In this context, it is not surprising that noisy, relatively broadband signals, like those present in natural environments, are among the most effective in stimulating the activity of the medial efferent system (104). The MOC system seems to stabilize active micromechanical properties in humans, as the MOC system elicits a reduction in the amplitude varibility of EOAEs (105). It is known that the olivocochlear neurons require 50 to 500 ms of stimulation before they respond. The medial system has a fast response and slow response within milliseconds and steady state response that remains constant for hours (106). It was shown that the ILD (interaural latency differences) are reduced, when contralateral noise is added, and what appears to be significantly correlated to the contralateral EOAE amplitude attenuation effect. These results support the hypothesis that MOS system plays a role in intensity discrimination in dichotic noise in humans (107).

**Efferent nerve response patterns:** The medial and the lateral efferent fibers possess different kinetics of transient outward currents, what seems to be responsible for the differences in firing properties. Both show spike trains and tonic patterns in response to injection of depolarizing currents at the resting membrane potential. However when the membrane is slightly hyperpolarized, lateral olivocochlear fibers show spike trains with a first long interspike interval, whereas medial olivocochlear neurons showed a spike train with a long latency to the first spike (108). The response adaptation of the medial efferent fibers is minimal compared to other auditory fibers. Sustained responses may enable the MOC system to produce sustained effects in the periphery, supporting a role for this efferent system during ongoing stimuli of long duration (109). Transection or disruption of the lateral efferent system compresses spontaneous rates of firing among auditory nerve fibers with an overall decrease in CAP of the cochlear nerve, whereas the nerve threshold sensitivity and N1 latencies are relatively unchanged (110), supporting the hypothesis that lateral olivocochlear neurons modulate single-unit auditory nerve activity (111, 112).

**Basilar membrane (BM) function and frequency selectivity regulation:** It is now commomly agreed on that some of the medial efferents effects are mediated via the cochlea’s mechanics, with the outer hair cells acting as the mechanical effector. A stimulation of the efferent bundle leads to an increase of the ampiltude of the microphonic waveforms, but no shape alteration in the cochlea. The impedance of the basolateral wall of the outer hair cells declines by about 50% and the vibration of the organ of Corti increases by about 20% at low frequencies in guinea pigs (113). Efferent nerve activation produces a decrease in the velocity of the basilar membrane amplitude for frequencies around the best frequency (BF, highest basilar membran velocity) at low stimulus levels with no or little effect for stimuli well below the BF. The olivocochlear bundle activation changes the gain of the voltage-dependent OHC motility such that BM velocity response near BF is decreased while increasing the response for tones well above BF (114). For tones near the charcteristic frequency (CF, equal to the frequency of the tone by definition for a pure tone of low level), a stimulation of the olivocochlear bundle tends to linearize the highly compressive displacement-level functions and to displace the steep low-level region toward higher intensities along the intensity axis by <27 dB SPL. This shift results in a desensitization of the tip of the BM displacement tuning curve that is sometimes associated with downward shifts in the tuning curve of <500 Hz. Thus the effect on the frequency tuning curve of the BM is very similar to the effect of olivocochlear bundle stimulation on the sensitivity and frequency tuning of afferent fibers and inner hair cells (115). It was shown that patients with an impaired efferent system have a reduced ability to focus attention in the frequency domain and detect signals at unexpected frequencies better than before (116). Crossed olivocochlear efferents reduce the receptor potentials on inner hair cells predominantly at the point of highest frequency selectivity. But they have a slight effect on resting membrane potentials. At high sound levels the receptor potentials are less reduced compared with lower intensities (117). Sectioning of the efferent bundle decreases frequency selectivity, as an enlargement of the tip segment of the CAP tuning curve can be found, and the Q10 dB value (10 dB above threshold) decreases by about 30% without any significant threshold change (118).

### Pathophysiological relationships

**Acoustic trauma:** The olivocochlear bundle is one of the main noise protective mechanisms of the cochlea. It was speculated that rather the medial efferent system evolved in the context of unmasking transient stimuli, rather than protecting the inner ear from intense noise levels, as significant selective pressure to segregate biologically relevant acoustic signals from irrelevant background noise might be expected (119). But it is still controversial if this capability is an evolutionary by-product, as protective effects start within traumatizing noise levels or lower noise levels and main function of the olivocochlear bundle consists of cochlear fine regulation (119, 120). The medial efferent system mainly provide the cochlear protection, but the lateral efferent system seems to contribute as well by protecting cochlear nerve dendrites from excitoxic effects of acoustic overexposure (110, 121, 122). The ability to evoke the protective effects is strongly dependent upon sound context, intensity, duration and frequency, and the latter might correlate with the cochlear innervation pattern described above (123, 124). Animals with a strong MOC reflex show less threshold shift after acoustic overstimulation than those with weak reflexes (125). In addition, it is possible that the variability of the MOC reflex strength has a genetic basis (126). De-efferented ears show an increase of the permanent threshold shift (PTS), the temporary threshold shift (TTS) and the outer hair cell loss after noise exposure (127). Overexpression of alpha9-Ach receptors in the outer hair cell in transgenic mice significantly reduces acoustic injury that causes either temporary or permanent damage, without changing preexposure cochlear sensitivity to low-or moderate level sound (128). It is interesting that regenerated nerve fibers in the noise-damaged chinchilla are only afferent and have no AchE staining (129).

Noise protective effect of low-level sounds or vibrations is known for a long time. MOC efferent terminals and outer hair cells are protected by sound conditioning preceeding the noise exposure (130). It could be shown that sound conditionig protects against the decrease of tyrosine hydroxylase (TH) immunolabeling by acoustic trauma and increases fiber staining for TH in the lateral superior olive and posterolateral periolivary nucleus, but not in the dorsal periolivary nucleus and lateral nucleus of the trapezoid body (131).

**Other auditory system stressors:** Contrastingly to noise, aminoglycosides show distinct differences regarding caused impairment of the efferent system. Hearing recovery takes substantially longer after aminoglycoside application than after sound damage. Different types of aminogycosides damage the olivocochlear bundle and inhibit the maxi-K^+^ channel in single isolated efferent nerve terminals with different intensities (132, 133). Neomycin inhibits the cochlear dopamine release dose-dependently, while gentamicin and kanamycin seem to be ineffective on it. After chronic application of neomycin the dopamine outflow did not change significantly, suggesting an adaptive process (134). There exists a rapid reversible dose-dependent elimination of the medial olivocochlear bundle function following single gentamicin injection with doses where no hair cell damage could be detected (135, 136). At low doses the fast response of the medial bundle is blocked and at higher levels the slow and steadystate response are blocked additionally (106). The inhibitory effect of gentamicin might be explained by non-competetive cholinergic inhibition of nicotinic acetylcholine receptors (nAChRs) at the level of the postsynaptic membrane in outer hair cells by displacing calcium from specific binding sites of nAChRs and alterating the cation current of outer hair cells (137).

**Tinnitus:** Importance of the efferent stimulus on tinnitus generation and manifestation at the brainstem level has been suggested by Jastreboff and Hazel who emphasized the connection with the reticular formation and Robertson *et al*. who pointed out the connection at the cochlear nucleus affecting the ascending auditory pathway seperately from influence upon the cochlea (138, 139). Various neurotransmitters might be involved in tinnitus generation. Carbachol as direct ACh agonist shows tinnitus improvement or disappearance for 12-72 hours after transtympanal application (140). In the presence of dynorphins, the excitatory neurotransmitter glutamate is enhanced. This results in an altered neural excitability and/or an altered discharge spectrum in (modiolar-oriented) type I neurons normally characterized by low rates of spontaneous discharge and relatively poor thresholds (141).

Medial efferent function measured by contralateral suppression is impaired in tinnitus and hyperacusis, but seems to be not affected in sensorineural hearing loss (142). There is a clear relationship between SOAEs and the efferent modulation of the cochlea. Modulation of the cochlear active mechanisms mainly takes place in the low-and mid- frequency regions correspond to the frequency range of SOAEs and medial efferent innervation patterns (143). An increased threshold for TEOAEs and an elevated prevalence with increased variability of SOAEs was shown in patients with past longterm noise exposure and resulting tinnitus compared with those without tinnitus (144). Tinnitus patients respond with loss of suppression in contralateral white noise stimulation resulting in increased TEOAEs, whereas healthy controls show a decline of TEOAEs (145).

**Other relationships:** The terminology Ried (retrocochlear inhibition efferent deafness syndrome) was proposed for sudden or rapidly progressive hearing loss, which is accompanied by tinnitus, and occasionally by dizziness, related to stressful situations, undergone by tense and perfectionist people who are unable to relax. An active efferent inhibition and neurotransmisson disorder was proposed (146) as well as in children with auditory processing disorders who complain of difficulties of understanding speech in the presence of background noise (147).

It was shown that myasthenia gravis reduces TEOAEs and DPOAEs and reversion takes place after application of a AChE inhibitor (148). The complete absence of contralateral suppression together with an absence of ABRs, MLDs and a nearly normal audiogram (auditory paradox) may reflect a lower brainstem disease like multiple sclerosis, Charcot-Marie-Tooth, Friedeich’s ataxia, primary neuropathies. Patients have severe impairment of speech comprehension particularly in noise and might be attributed to afferent nerve desynchronisation and disconnection to the efferent system (149).

It could be shown that autistic children seem to have an impaired medial efferent function in the left ear and children with learning diorders mainly have a reduced function of the medial efferent system in the right ear (150). The absence of the superior olivary complex occuring in autism might contribute to the disconnection from the outer world that characterizes this syndrome (151). More than half of the patients with autisic disorder have abnormalities in auditory brain stem responses (ABR). The most common findings are prolongation of wave V and of I-V interpeak latency (IPL) (152). Generation of waves IV and V in human has been ascribed to the brainstem at the level of the superior olivary complex (8).

In addition, hyperacusis like in Williams syndrome was attributed to loss of inhibitory modulation to efferent sensory input to the cochlea (153). Loss of medial olivocochlear suppressive function may play a role in the development of presbyacusis in clinical cases and animal models as it could be shown that contralateral suppression declines at low-frequencies in old aged animals. DPOAEs in mice decreased with age in a way similar to humans (154).

## CONCLUSION

It is commonly agreed on that the efferent system possesses relationship to distinct pathologies of the auditory system and holds key role in noise protection of the auditory system on the one hand and fine regulation of hearing perception including mediation of selective attention, improvement of signal to noise ratio, adaptation and frequency selectivity on the other hand. The lateral and medial efferent system together form the basis for localization of a sound stimulus and enable us to function in a three-dimensional auditory world. Various neurotransmitters are involved in the subtle mechanisms of fine regulation of the efferent system ensuring above mentioned mechanisms. Distinct functional differences of the two systems are understood, but still insufficiently for preventive and therapeutic modification of the efferent system.

## ABBREVIATION

ABR: auditory brain stem response
ACh: acetylcholine
AChe: acetylcholinesterase
ATP: adenosine triphosphate
AVCN: anteroventral cochlear nucleus
BF: best frequency
BM: basilar membrane
CAP: compound action potential
CF: characteristic frequency
CGRP: calcitonin gene related peptide
DCN: dorsal cochlear nucleus
DPOAEs: distortion product otoacoustic emissions
EOAEs: evoked otoacoustic emissions
GABA: gamma aminobutyric acid
GTP: guanosine triphosphate
IHC: inner hair cell
ILD: interaural latency differences
IPL: interpeak latency
Isk: small conductance channels
LSO: lateral superior olive
nAChR: nicotinic-like ACh-receptor
NO: nitric oxide
MSO: medial superior olive
OAEs: otoacoustic emissions
OHC: outer hair cell
PTS: permanent threshold shift
PVCN: posteroventral cochlear nucleus
SEOAEs: spontaneous otoacoustic emissions
SPL: sound pressure level
TEOAEs: transient evoked otoacoustic emission
TH: tyrosine hydroxylase
TTS: temporary threshold shift
VCN: ventral cochlear nucleus
